# An Underutilized Food “Miwu”: Diet History, Nutritional Evaluations, and Countermeasures for Industrial Development

**DOI:** 10.3390/foods12071385

**Published:** 2023-03-24

**Authors:** Jinpeng Zou, Jiayi Wang, Kai Hou, Fang Wang, Shiwen Su, Wenjing Xue, Wei Wu, Ni Yang, Xuan Du

**Affiliations:** 1 College of Management, Sichuan Agricultural University, Chengdu 611130, China; 2 College of Agronomy, Sichuan Agricultural University, Chengdu 611130, China; 3 Division of Food, Nutrition and Dietetics, University of Nottingham, Sutton Bonington Campus, Loughborough LE12 5RD, UK

**Keywords:** diet history, health function, industrial development, Miwu, *Ligusticum chuanxiong* Hort., medicine and food homology, nutritional composition, safety evaluation, SWOT analysis

## Abstract

About 10 major crops basically feed the world. In fact, there are still a large number of plants that have not been fully explored and utilized because they have been ignored by the market and research. The expansion of food sources in various countries plays an important role in maintaining food security and nutrition security in the world. Miwu is the aerial part of the medicinal plant Rhizoma Chuanxiong belonging to a traditional local characteristic food raw material. Its edible value is still little known. Through textual research, component determination, literature survey, field research, and SWOT analysis, this paper has a comprehensive understanding of Miwu’s diet history, chemical components, safety risks, and industrial development status. It is found that Miwu has been eaten for 800 years, is rich in nutrients and active ingredients, and has no acute toxicity. In addition, the current industrial development of Miwu has significant advantages and many challenges. To sum up, Miwu is a potentially underutilized food raw material. This paper also provides countermeasures for the industrialized development of Miwu, which will provide a milestone reference for the future utilization and development of Miwu.

## 1. Introduction

*Ligusticum chuanxiong* Hort. (CX), a medicine food homology plant of Umbelliferae, whose roots, *Chuanxiong Rhizoma* (CXR), is a traditional southeast Asian herb that is used clinically to treat chest pain, headache, irregular menstruation, etc. [1,2]. *Chinese Pharmacopoeia* (Ch.P. 2020) published 1607 traditional Chinese medicine preparations, 246 of which contain CXR, accounting for 15.3% of the total and including Suxiao Jiuxin pills, Siwu mixture, etc. [3]. In 2002, CXR was approved to be a health food and could be used to produce health foods by the National Health Commission of the PRC (NHC). However, the stems and leaves of CX (Miwu, “蘼芜” in Chinese), the non-medicinal part, has been ignored for a long time, causing Miwu to become an agricultural waste [2]. With the continuous increase in the planting scale of CX, governments and growers started to see its great potential as a food material [4]. In order to facilitate and standardize the international trade of CX, our group considered not only the relevant indicators of CXR but also the indicators of Miwu when we drafted ISO/TC249 Traditional Chinese Medicine—*Ligusticum Chuanxiong* Rhizome (ISO/CD 8071) [5].

At present, Miwu has a huge amount of biological resources because it (the stems and leaves of CX) accounts for more than 75% of the fresh weight of CX, which could sprout tumble twice in the growth period. Meanwhile, according to a survey, the planting area of CX only in Chengdu, Sichuan reached 7000 ha, and the annual production of Miwu could reach more than 50 million tons [6]. However, the amount of Miwu decaying in the field may pose a threat to environment. Therefore, if Miwu can be effectively developed and utilized it will not only reduce the environmental pressure but also bring economic “spillover effect” to growers. In fact, Miwu has been an edible vegetable in *Daodi* areas (genuine producing area, the main CX optimal production areas) such as Chengdu, Meishan, and Deyang in Sichuan province for a long time [7,8]. In these places, Miwu have been gradually developed into different products, e.g., healthy tea, flavoring, craft, animal feed, and organic fertilizer [7,8,9,10,11,12,13,14,15]. In the Chinese diet, Miwu is used for tea, salad, and soup or stir-fried, etc., and has been eaten for nearly a thousand years. Although the consumption of Miwu has been continuous in Chinese history, its consumption has gradually decreased in modern times. One important reason may be that it has gradually become a food in the *Daodi* area, although it is also changing with the climate [16].

Nevertheless, research on Miwu is currently scattered and rare, which leads to a lack of scientific and systematic research of its edible history, chemical composition, nutritional value, potential risk, and its industrial condition. Therefore, the edible history, nutritional components, chemical components, pharmacological effects, edible risks, and industrial development status of this plant are comprehensively studied in this paper, which has great potential value for future interdisciplinary research on Miwu and other underutilized crops or lesser-known foods. Section 2 conducts ethnobotany research; afterwards, Section 3 and Section 4 exert the textual research and review on Miwu’s diet history at home and abroad. Principal components, pharmacological effects, and edible risk are presented in Section 5 and Section 6. Section 7 analyses Miwu’s industrial condition by SWOT analysis. In the end, Section 8 concludes the paper. This paper can also provide some references for formulating international standards of CX.

## 2. Ethnobotany

**Figure 1:**
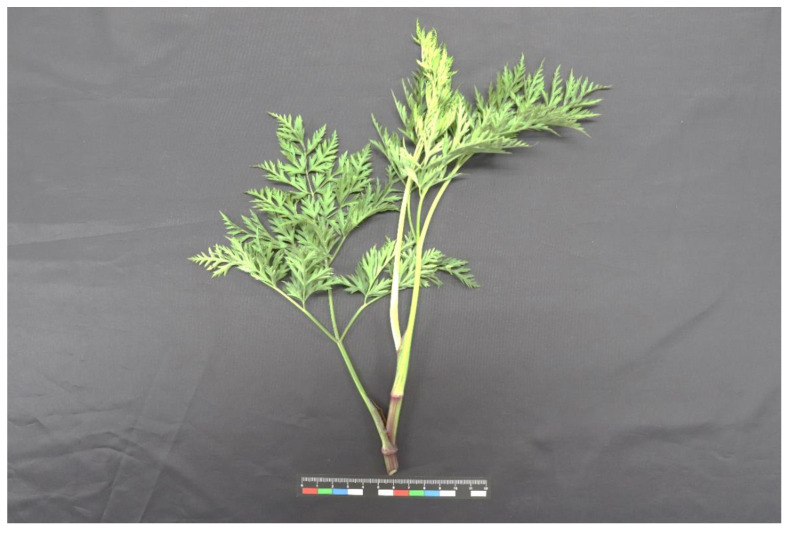
Botanical appearance of Miwu.

CX’s height is 40–60 cm and its stem is upright and cylindrical with longitudinal stripes and many branches on the upper part. The outline of the leaf is an oval triangle with a length of 12–15 cm and a width of 10–15 cm. It is pinnately divided into three to four times and three times. There are 4–5 pairs of pinnae, which are ovate–lanceolate with a length of 6–7 cm and a width of 5–6 cm. The last lobe is linear–lanceolate to oblong, with a length of 2–5 mm and a width of 1–2 mm and a small tip at the top and only sparse veins. The upper leaves of the stem are gradually simplified [17] (as shown in Figure 1).

## 3. The History of Using Miwu as Food in China and Other Countries

### 3.1. The History of Using Miwu as Food in China

It can be seen from the above that since the Han dynasty, Miwu uniformly referred to the tender stems and leaves of CX. Therefore, the literature on Miwu’s consumption after the Han dynasty is of great value for researching the edible history of Miwu. “Yuefu poetry in Han Dynasty” recorded: “Picking wild Miwu on the hill when she goes up the mountain, meeting her husband when she goes down hill” [18]. The Tang dynasty has a verse: “She carries baskets of red leaves to pick Miwu for a whole day”, which vividly depicts the scene of the ancient picking of Miwu that is used as accessories or ingredients [18]. Miwu is clearly recorded as a food material since the Song dynasty. “Illustration Classics for Materia Medica” in the Song dynasty recorded that “people in Jiangdong and Shuchuan picked Miwu to make tea which can make you refreshed”. Han Qi, the prime minister during the Northern Song dynasty, wrote a poem to describe the refreshed feeling of after drinking the tea made of Miwu [19]. In addition, Miwu is not only used to make tea but also used for daily food therapy. ”Lv Chan Yan Materia Medica” records that “Miwu tea tasted spicy, it is non-toxic and could treat headache”. Ancient Wang Jie enjoyed chewing Miwu followed by drinking tar after every meal. In addition to records of the *materia medica*, there are also records of food history in lyrics and poems. Song Qi’s “Brief Introduction of Featured products in Chengdu” published in the Song dynasty recorded the planting and eating history of Miwu in Chengdu: “People try to plant them in the garden, some people pick and muddled them and before mixed them in the soup”. In other words, Miwu could not only be used to make tea but also be used in soup porridge. In the Song dynasty, Fan Chengda’s book “Wu Chuan Lu” recorded that Taoists in Qingcheng Mountain planted CX on the mountain, indicating that artificial planting of CX was very common. It is recorded in the lines of the famous ancient poet Lu You; it was popular to drink Miwu tea in the spring after meals in his time. In the Southern Song dynasty, Zhang Zi’ poem showed his preference for Miwu, “The taste of the soup with Miwu is even better than any other tea”. The poem of Su Shi recorded the experience of picking and eating Miwu “Although I’m full, I’m still interested in finding Miwu in the grassland”. From 1016 to 1848, there are many historical books to record the ways to have Miwu (as shown in Table 1). People did not stop eating Miwu because of the change of dynasties, and they cooked it in various ways. To sum up, Miwu have been used as a food material (tea, soup, etc.) since at least the Northern Song dynasty; therefore, it has been used as a food for more than 800 years.

**Figure 2:**
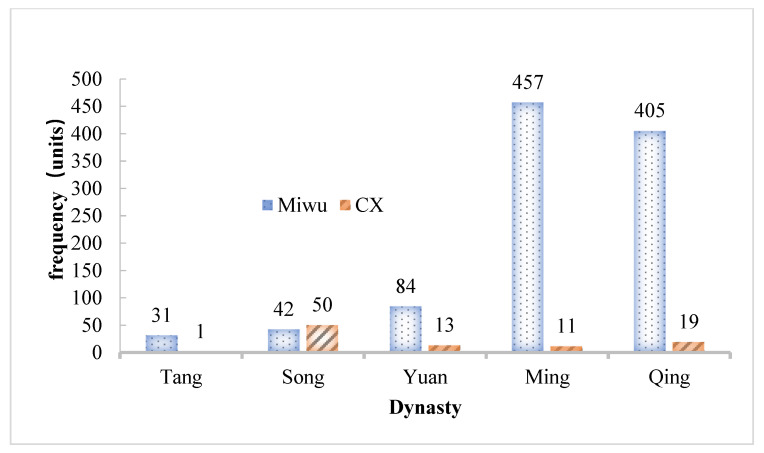
The frequency of Miwu and CX in ancient Chinese classical literature. Tang 618–907 AD, Song 960–1279 AD, Yuan 1271–1368 AD, Ming 1368–1683 AD, and Qing 1683–1840 AD.

According to the statistics of the China Poetry Website, the number of poems including the term “Miwu” gradually increased from 31 in the Tang dynasty to 457 in the Qing dynasty (shown in Figure 2) [20]. In other words, Miwu began to play a more and more important role in the catering of ancient people. Meanwhile, there were also various ways to cook Miwu. *Materia Medica for Famine Relief*, published in the Ming dynasty, is the earliest monograph on edible plants for famine relief in the world and still has practical value at the present time. It recorded that “Miwu could be fried for the food in the Great Famine”. In addition to decoction, frying, and baking, many records reported that Miwu was an ancient raw material for making tea or soup. According to the field survey of our team in Pengzhou City and Dujiangyan City in Sichuan Province, which have been *Daodi* areas for a long time, we find that the tender Miwu became a seasonal vegetable in the rural markets every spring. A cold mix of Miwu, Miwu salads, stir-fried Miwu, stewed chicken with Miwu, Miwu tea, and Miwu noodles have become the local specialty foods. With the booming development of rural tourism, they are a lot of people’s preferences [21,22]. Although a larger market is waiting to be developed, the current market regulation could not provide this possibility.

### 3.2. The History of Using Miwu as Food in Other Countries

In Japan and South Korea, Miwu is considered a healthy vegetable or seasoning and is popularly used in salads and other meals; consumption of it is increasing [23,24]. In 2021, the first batches of fresh Miwu were exported from Chengdu to South Korea as vegetables [25]. In addition, CX was listed in the edible catalogue database of the British Future Plant Network (Plants For A Future: A Resource and Information Centre for Edible and other useful plants) [26]. This database suggests that the edible part of CX is its leaves and the tender stems.

## 4. Chemical Components

### 4.1. Nutritional Components

At present, there are few research reports on the nutritional components of Miwu. In order to accurately judge the nutritional characteristics of the stems and leaves of Rhizoma Chuanxiong, the nutritional components of Miwu produced in Aoping Town, Pengzhou City were detected. The results are shown in Table 2. Miwu is rich in carbohydrates, dietary fiber, protein, iron, zinc, niacin, and β-carotene but lacked Se, Vitamin A, Vitamin B1, and folic acid. The amount of the dietary fiber in Miwu is 5900 mg/100 g, which is 4.2 times that of celery, whereas the sodium content of celery is 62.5 times higher than Miwu.

The high dietary fiber content and good nerve and vascular protection give Miwu the reputation of a future food [28,29]. The plant is considered to be suitable for future space travel.

### 4.2. Active Components

The rhizome, leaves, and fibrous roots of *L. chuanxiong* Hort. are similar in composition but have different contents [30]. 

The main active components of Miwu include phthalide and its dimer, alkaloid, organic acid, terpenes and its enols, and others. Part of principal components previously found in Miwu are summarized in the Table 3, although it is not possible to include them all.

#### 4.2.1. Phthalides and Its Dimer

**Figure 3:**
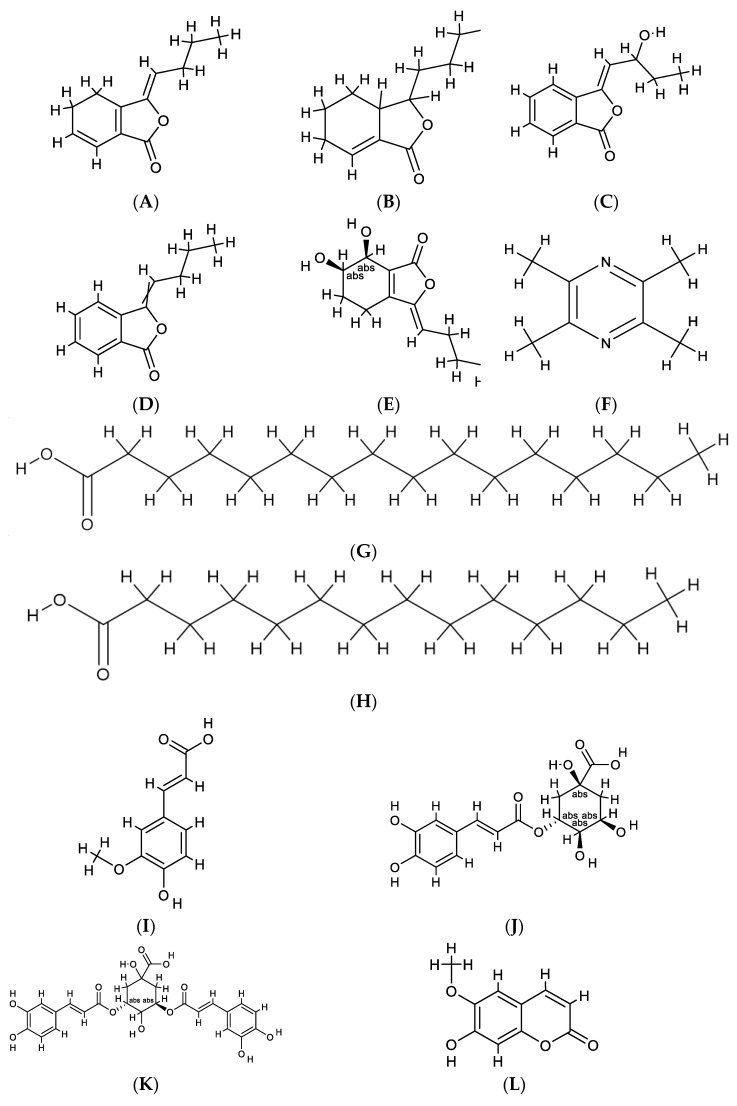

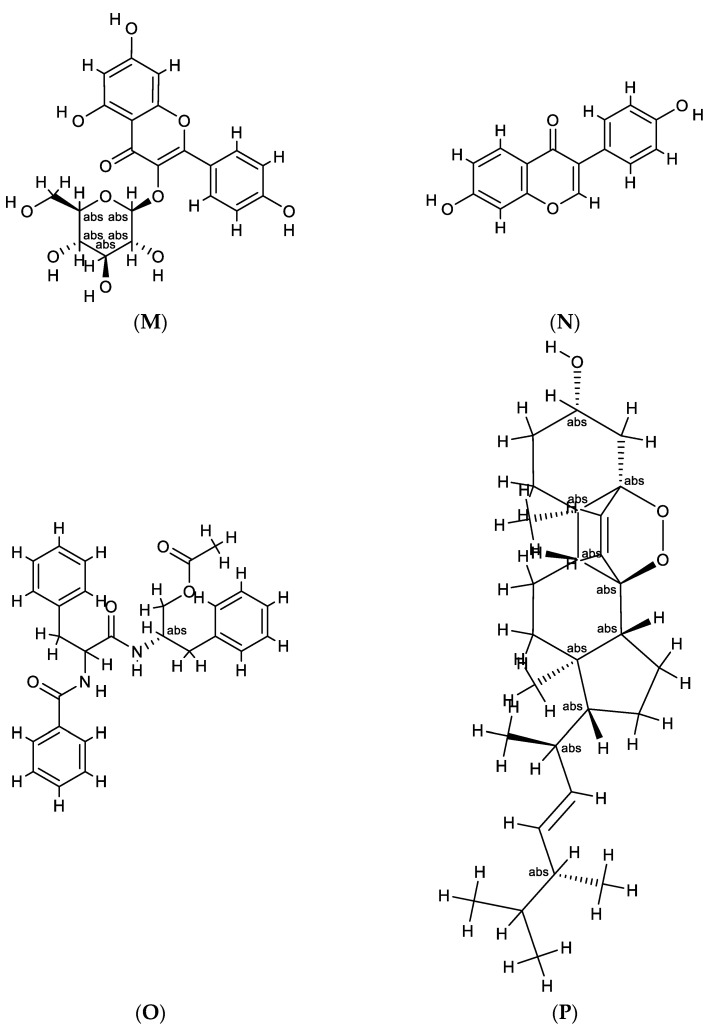
Chemical structure of the principal chemical compounds in Miwu. (**A**) Z-Ligustilide. (**B**) Sedanolide. (**C**) Senkyunolide-E. (**D**) Z-3-Butylidene phthalide. (**E**) Senkyunolide H. (**F**) Tetramethylpyrazine. (**G**) N-Hexadecanoic acid. (**H**) Tetradecanoic acid. (**I**) Ferulic acid. (**J**) Chlorogenic acid. (**K**) 3,5-Dicaffeoyl quinic acid. (**L**) Scopoletin. (**M**) Astragalin. (**N**) Daidzein. (**O**) Aurantiamide acetate. (**P**) Ergosterol peroxide.

Phthalides, a class of ester derivatives bearing a benzene ring fused to a five-membered lactone ring, belong to o-hydroxymethylbenzoic acid lactone [42]. It is characterized by small molecular polarity, volatility, and instability and is a typical representative component of volatile oil from CX [43]. The content of phthalides in fresh leaves is the highest, especially Z-ligustilide (Figure 3A) [37]. In 1988, Huang Yuanzheng and Pu Fading detected Z-ligustilide and sedanolide (Figure 3B) in the essential oil of Miwu [40]. Tang Fei et al. isolated senkyunolide-E (Figure 3C), neoligustilide, and Z-3-butylidene-phthalide (see Figure 3D) from Miwu for the first time in 2020 [44]. The same year, Liu Juanru et al. extracted the volatile oil by steam distillation and analyzed and identified the chemical components of the volatile oil by GC-MS. Then, senkyunolide H (Figure 3E) was detected in Miwu. In addition, some new phthalides and their derivatives have also been discovered [33].

#### 4.2.2. Alkaloid

The alkaloids in CX are small molecular compounds with low content. Among them, tetramethylpyrazine (Figure 3F) is the most widely reported in modern research [45]. In 1977, Chinese researchers found tetramethylpyrazine (TMP) in CXR for the first time [46]. Then, several alkaloids were continuously isolated from CX. To date, domestic and foreign scholars have isolated 20 alkaloid compounds from CX [47,48,49]. As alkaloids are important pharmacodynamic substances of CX, there are many assays for the content of its root. However, Rui Hekai et al. also detected the presence of TMP in Miwu [50].

#### 4.2.3. Phenolic Acid

Phenolic acids, characterized by the presence of one or more hydroxyl groups attached to an aromatic ring structure, were also detected in Miwu. Up to now, four phenolic acids have been detected in Miwu, namely caffeic acid tetradecanoic acid, ferulic acid, chlorogenic acid, and 3,5-dicaffeoyl quinic acid (as shown in Figure 3G–K). Yijinhai et al. determined that different parts of CX contained ferulic acid by RP-HPLC, with higher contents in rhizome and fibrous roots and lower content in Miwu [38]. In 2018, chlorogenic acid and 3,5-dicaffeoyl quinic acid were detected by Wu Yan et al. [39].

#### 4.2.4. Others

Nie et al. studied the chemical components of the aerial parts of CX in 2011 using a variety of separation methods (silica gel column chromatography, ODS column chromatography, sephadex LH-20) and detected scopoletin, astragalin, daidzein, aurantiamide acetate, and ergosterol peroxide [35] (see Figure 3L–P). At present, the content determination of polysaccharides in Miwu has not been studied.

## 5. Pharmacological Activities

### 5.1. Phthalide and Its Dimer

Phthalides have various biological activities and pharmacological effects, such as in the prevention and treatment of cerebrovascular diseases and neuroprotection, anti-tumor, antibacterial, vasodilator, anticoagulant, insecticidal, anti-inflammatory, and anti-virus activities [51]. Ran et al. reviewed the anti-oral cancer mechanism of drugs containing *Ligusticum chuanxiong*, and they believed that butylidenephthalide and its derivatives could effectively induce apoptosis of oral cancer cells [50].

#### 5.1.1. Anti-Alzheimer’s Disease

Phthalide monomers have significant pharmacological activities against cardiovascular and cerebrovascular diseases. For example, Z-ligustilide has a potential therapeutic effect in Aβ1-40-induced PC-12 cell injury in an Alzheimer’s disease model [51]. In addition, relevant studies have also shown that Z-ligustilide has significant neuroprotective effects [52]. 

#### 5.1.2. Antineoplastic Activity

It was found that Z-ligustilide significantly inhibited the proliferation of L1210 and K562 tumor cells, with IC50s of (2.27 ± 0.10) and (4.78 ± 0.18 μ/mol·L^−1^), respectively [53].

#### 5.1.3. Vasodilator

Phthalides can penetrate the blood–brain barrier so have potential activities in the prevention and treatment of cerebrovascular diseases. Z-Ligustilide is considered to be the main active compound among these phthalides. Studies show that it has vasodilation, antiasthmatic, antiplatelet aggregation, analgesia, antithrombosis, and antiproliferation effects [54,55,56]. The main metabolites of ligustilide in vivo were senkyunolide I, senkyunolide H, etc. [57]. Senkyunolide I was able to modulate placental growth factor (PIGF) to control the production of endothelial cells of microvessels, significantly promoting the formation of luminal structures by microvascular endothelial cells and thereby acting as a vasodilator [58]. Moreover, some studies have shown that senkyunolide-H can inhibit the proliferation of smooth muscle cells and induce the expression of hemeoxygenase-1 [59]. 

### 5.2. Alkaloid

#### 5.2.1. Anti-Cerebral Ischemia

Tetramethylpyrazine can improve microcirculation, increase cerebral cortex blood flow, and promote the recovery of neural function. It can protect brain tissue from ischemia and hypoxia injury by regulating the expression of apoptotic and pro-apoptotic genes [60,61]. 

#### 5.2.2. Heart Protection

Tetramethylpyrazine has the effect of protecting ischemic myocardium. It can stabilize the content of Ca^2+^ in mitochondria, increase the activity of mitochondrial Ca^2+^ ATPase, protect the structure and function of mitochondria, reduce the damage of mitochondrial function, and play a pre-protective role in cardiac function [62,63]. 

#### 5.2.3. Protect Optic Nerve

Tetramethylpyrazine can significantly alleviate the oxidative stress damage of hydrogen peroxide on retinal neurons and glial cells and up regulate the expression level of tubulin-2 and neuroprotective peptide in the nervous system, which are closely related to cell survival [60]. At the same time, tetramethylpyrazine has the effects of protecting the liver and kidneys, relieving asthma, and has anti-tumor activity [61,62,63].

### 5.3. Organic Acids

#### 5.3.1. Antioxidant Stress

Caffeic acid and chlorogenic acid can inhibit the key enzymes related to the lipid peroxidation of rat brain caused by Alzheimer’s disease and some oxidants, possibly by inhibiting the activities of acetylcholinesterase and cholinesterase and thus slowing down the decomposition of acetylcholine and butylcholine in the brain to prevent oxidative neurodegeneration [64].

#### 5.3.2. Antithrombosis

Chlorogenic acid degraded blood clots, which was similar to thrombolytic agents in preventing and treating thrombosis, and restrained the enzymatic activity of procoagulant proteases, thrombin, activated factor X (FXa), and activated factor XIII (FXIIIa). Research showed that chlorogenic acid inhibited platelet activation by the following mechanism of action: A2A receptor/adenylate cyclase/cAMP/PKA activation and, consequently, suppression of activation of the GPIIb/IIIa receptor and platelet secretion [65].

### 5.4. Others

Astragaloside is a kind of natural flavonoid active compound that can be isolated from a variety of medicinal plants and has extensive pharmacological properties. It has anti-diabetes, anti-obesity, anti-osteoporosis, anti-fibrosis, and other effects [66,67,68,69].

According to previous research, the antidepressant-like effect of scopoletin was dependent on the interaction with the serotonergic (5-HT) receptors, which may be implicated in the regulation of mood disorders, dopaminergic (D1 and D2 receptors) systems, and noradrenergic (α1-and α2-adrenoceptor) systems. These are thought to be the foundation of some of the antidepressant-like responses of drugs in behavioral models of antidepressant activity [58,70]. Studies show that aurantiamide acetate has anti-neuroinflammation, anti-virus effects, etc., [71,72].

## 6. Food Safety Risk

A large number of studies have uncovered that the components of the aerial part and the underground part of CX are similar but their expressions are different [24,36,37,73]. Excessive Cd in CXR has become a common phenomenon; however, heavy metals are mostly enriched in rhizomes and the content of Cd in the aerial part is very low [74]. Some scholars argued that the aqueous extract of CX had no mutagenicity, and the bacterial reverse mutation assay and in vivo mammalian micronucleus study were negative [24,75,76]. In a famous book, *Mingyi Bielu*, “Supplementary Records of Famous Physicians”, a famous Chinese herbal treatise published in 221 AD, Miwu was deemed as avirulent. A preliminary study on food safety showed that the maximum tolerance of mice for the aerial part of CX was 84 g/kg, which was more than 500 times the daily adult dosage. Based on the long-term diet habit, we hold the view that Miwu has no acute toxicity and chronic toxicity within a limited dose, whereas there are still no carcinogenicity tests, teratogenicity tests, and mutagenicity tests and subchronic toxicity tests have yet been reported [7,77].

In China, CX is officially recognized as a health food raw material and is included in the feed raw material catalog [78,79]. A recent article completed by the Sichuan Agricultural University shows that the acute toxicity of the leaves and rhizome on *Caenorhabditis elegans* could be reflected by the viability. A range of concentrations between 250 and 1000 g/mL of leaves and rhizome of CX were found to be safe for *C. elegans*. These concentrations did not affect worm viability after 24 h when compared with the control group; the leaf and root of CX are safe as medicinal and edible plant foods [30,80,81]. Moreover, it has been used in Japanese, Korean, and other traditional medicine for over 2000 years. Currently, it is mostly cultivated and has high safety and low side effects [30].

## 7. Industrial Status of Miwu

In addition to further and more rigorous toxicological research, from the above analysis of diet history and nutritional value it can basically be judged that Miwu is an edible local traditional food. At present, the key factors restricting the development of its industrialization are not the Miwu itself and technology limitations but government supervision and the market. Expanding the Miwu industry and realizing the sustainable development of CX requires sorting out the key issues affecting the industrial development of Miwu to release the huge economic and economic and social value. Therefore, this paper tries to find out the key factors that affect Miwu’s value enhancement through 6-month field research in 2022 and SWOT analysis to analyze and judge the current basic situation of the Miwu industry and finally put forward countermeasures and suggestions for future development.

Ricardo formed the theory of comparative advantage based on the differences in production costs and factor prices that may result from differences in natural resource endowment and production factors [82,83,84,85]. The theory was originally used to explain the global division of labor in international trade to improve the production efficiency of each country. This theory is also applicable to explaining the development of inter-regional agricultural characteristic industries. In addition to traditional production factors such as labor force, capital, and land, historical, cultural, and natural elements in each region can be considered [1,84]. According to Michael Porter, an industrial chain is an enterprise group structure composed of a series of interdependent upstream and downstream chains that are involved in serving specific needs or producing specific products [1,20,86]. Generally, the coordination and optimization of the industrial chain could be realized through five aspects: space chain, enterprise chain, technology chain, product chain, and value chain. The optimization of the industrial value chain mainly considers three key dimensions: product value, production efficiency, and value realization conditions [87,88]. Product value mainly includes three dimensions: market value, service value, and image value [27,89]. The improvement of market value mainly depends on innovation ability, that is, the ability to develop new products or services to satisfy consumers; service value refers to the degree to which additional service capabilities of products satisfy consumers; image value refers to the impact of intangible forces represented by brand influence on consumers’ degrees of satisfaction. The improvement of production efficiency mainly considers five dimensions: capital, technology, human capital, organizational form, and policy support. Through the adjustment and optimization of these five dimensions, transaction costs in the middle of the industrial chain can be reduced and the speed and quality of information exchange can be improved [90,91,92]. The realization of value is mainly considered from the dimension of the market, is supported by the sales environment, and the core is the marketing ability [87,88].

**Figure 4:**
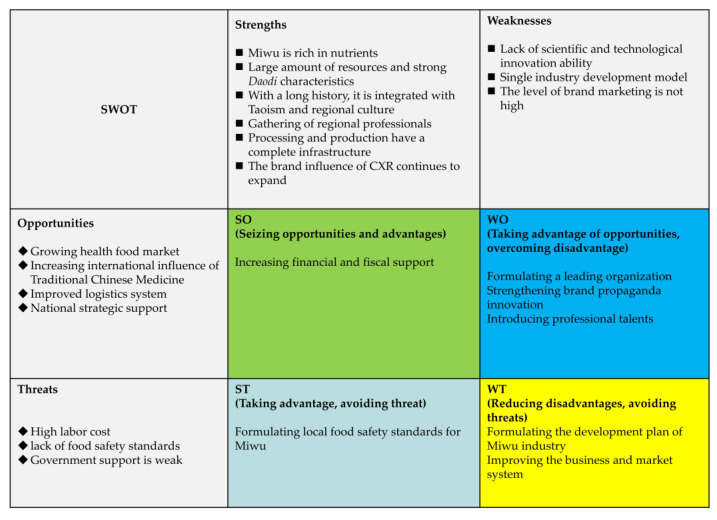
SWOT analysis of the Miwu industry.

The results of the SWOT analysis are shown in the Figure 4.

### 7.1. Strengths

As a Daodi herb medicine, Miwu itself combines unique natural resource endowment, cultural endowment, and health endowment, which is the innate foundation and advantage for the development of Miwu industries. At the same time, its best cultural area is located in the Chengdu plain, which has convenient transportation and low logistics costs. Therefore, the industry has industrial competitive advantages in natural resource endowment, traditional culture, and geographical location. The local industry–university research cooperation is very close. There are many universities and scientific research institutions in Chengdu, such as Sichuan Agricultural University, Chengdu University of Traditional Chinese Medicine, Sichuan University, etc. They maintain close industry–university research cooperation with the Miwu industry. The local area has a complete foundation for food and drug development. There are more than 20 local pharmaceutical manufacturers, among which nine have single products with annual sales exceeding 100 million yuan, such as Shugan Jieyu capsules of Chengdu Jishengtang Pharmaceutical Co., Ltd., Sichuan Province, China and Chengdu antiviral granules of Everbright Pharmaceutical Co., Ltd., Sichuan Province, China. The influence of the CXR brand is gradually expanding. In 2003, CXR from Chengdu was registered as a trademark, and in 2015 it was registered and protected as a geographical indication of agricultural products.

### 7.2. Weaknesses

First of all, the collection of Miwu relies on small farmers and enterprises to independently collect, develop, and sell Miwu. The basic advantages of the traditional Chinese medicine industry have not yet been used to strengthen the business cooperation of various business entities such as farmers, cooperatives, and pharmaceutical companies. Secondly, the processing technology level of Miwu is relatively low and intensive processing has not yet been realized. Chengdu Hushi Houmen Biotechnology Co., Ltd., Sichuan Province, China and Chengdu Jiangmao Pharmaceutical Development Co., Ltd., Sichuan Province, China produce some products such as Miwu tea, Miwu noodles, and Miwu biscuits; meanwhile, some catering companies use Miwu to make special meals and animal feed.

However, these products or services are limited to rough processing. In addition, there is rare government or social capital to invest in innovative technology for processing Miwu, which is the reason why the technical level is low, the innovation ability is weak, and the products are rough. Furthermore, the correlation between the planting industry and the food processing industry is weak, resulting in a low agglomeration effect. At present, there are still several industry associations and well-known enterprises to play a leading role. Therefore, the industry does not have enough ability to resist risks. In addition, due to the long-term publicity of CXR over Miwu in the past, people generally do not understand the diet and health value of Miwu. Moreover, the marketing and promotion are mainly carried out locally, whereas the promotion is limited to the form of news reports and word of mouth, leading to a small market.

### 7.3. Opportunities

First of all, as China’s per capita income continues to rise, people’s demand for food will have higher requirements [93]. Due to the special health effects, many medicinal plants have become food raw materials (See Table S1 in the Supplementary Material for some representative examples); they have a potentially huge market. As a local “healthy” vegetable, Miwu has the potential to meet the needs of current domestic consumption upgrades. Second, according to the statistics of the World Health Organization, because of the unique therapeutic effect of traditional Chinese medicine on coronavirus, it has been highly recognized by the international community, and the international influence of traditional Chinese medicine is also increasing [94,95]. Third, Miwu has multiple attributes of medicinal materials, agricultural waste, and local specialty food, so its industrialization development plays an important role in improving food and nutritional security and maintaining cultural value and common prosperity. According to the results of field research, the market price of Miwu was about 10 yuan/kg in 2022 and the annual output of young leaves of Miwu is about 7500 kg/ha, which can increase the income of growers by about 75,000 yuan/ha. Furthermore, there are many well-known pharmaceutical distribution companies in the *Daodi* areas, such as Chinese herbal medicine Tiandi, Bencaotang, and Hehuachi Pharmaceutical. Last but not least, these *Daodi* areas are also important vegetable production and distribution centers in the west of Sichuan and have a correspondingly complete sales network.

### 7.4. Threats

As a fresh vegetable and food ingredient, the production of Miwu requires a careful selection of fresh leaves and stems based on CX. Field research shows that picking Miwu requires more labor costs and time. Calculated by collecting 15,000 kg/ha Miwu, the current labor cost is about 67,500 yuan. Considering the transportation cost and wastage, the profit margin for Miwu is relatively low. At the same time, since products related to Miwu are in the introduction stage, a large amount of capital is required to invest in product development and sales, so the production cost is relatively high. At the same time, the government has not clearly approved it as a legal food raw material and there are no local food safety standard. Moreover, the local area has not been able to incorporate Miwu into the industrial development plan of CX. In other words, *Daodi* areas lack the goal and direction of developing Miwu.

### 7.5. Countermeasures to Develop the Miwu Industry

#### 7.5.1. Formulating Local Food Safety Standards for Miwu

**Figure 5:**
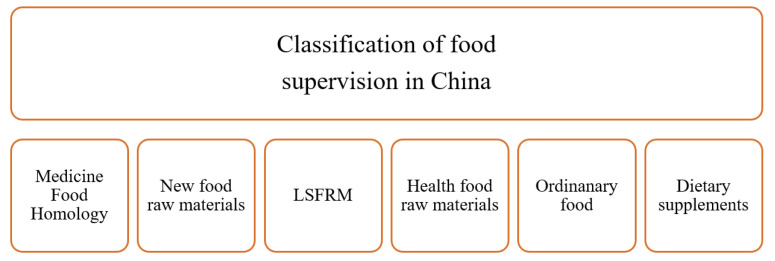
NHC’s classification of different types of food regulation.

Different types of food are regulated differently in China, as shown in the Figure 5. There are two ways for the non-medicinal parts of herbal medications to become legal food raw materials. One way is to register new food raw materials and another is to apply to be a local special food raw material (LSFRM). The LSFRMs are those raw materials that have been used for food production for more than 30 years. Actually, LSFRMs are as equally regulated as ordinary food but can be sold locally. Overall, a cost-effective pathway to enlarge the market of local traditional edible plants is to make them become an LSFRM. In China, many materials from the non-medical part of herb medicines in the Ch.P. have been declared as LSFRMs, e.g., *Cynanchum auriculatum* Royle ex Wight [96,97], *Eucommia ulmoides* Oliv [98], pine pollen [99], *Forsythia suspensa* [100], and Radix *Angelicae Dahuricae* [101]. Therefore, as a material that has been used in food for more than 30 years, Miwu has great potential to enter the list of LSFRMs.

#### 7.5.2. Formulating the Development Plan of the Miwu Industry

The government should incorporate the development of CXR and Miwu into unified industrial planning and design and consider the endowment advantages of each part of CX as a whole to effectively utilize production factors such as land, capital, and labor. For example, the original CXR processing equipment could be adjusted to realize the joint processing of the two parts. At the same time, it is necessary to introduce more products and services that integrate with rural tourism.

#### 7.5.3. Formulating a Leading Organization

The *Daodi* area needs to establish a communication platform among relevant government departments, the government, enterprises, and experts in planting, food safety risk assessment, and food processing and hold regular symposiums to gradually promote industrial development.

#### 7.5.4. Introducing Professional Talents

In order to improve the technological innovation capability of *Daodi* regions, it is necessary to give full play to the professional expertise of interdisciplinary talents. For example, Sichuan Agricultural University can give full play to its advantages in the breeding of CX and crop production to improve the yield and quality of Miwu; Xihua University can leverage its advantages in the development of intelligent agricultural machinery for Miwu to save labor costs and improve production efficiency; and Chengdu University of Traditional Chinese Medicine can give full play to its advantages in the field of medicine to extend Miwu’s industrial chain and increase its value.

#### 7.5.5. Increasing Financial and Fiscal Support

A system composed of finance and taxation is a key support for the development of modern characteristic industries. First, *Daodi* areas should set up a special fund for the development of the Miwu industry while encouraging social capital investment and gradually establishing a long-term mechanism for financial support. Second, the local government should expand financial subsidies for operating organizations that carry out recycling of Miwu and pilot agricultural machinery subsidies, insurance subsidies, etc. Third, regulating agencies could also provide training and consulting services for farmers. Fourth, corporate income tax reduction and exemption for relevant enterprises and cooperatives can be part of the government’s consideration. At the same time, credit, insurance, and financing support for relevant industrial chains should be strengthened.

#### 7.5.6. Strengthening Brand Propaganda Innovation

Based on the original brand influence of CXR and the concept of “Medicine and Food Homology”, joint efforts from the government, enterprises, and all sectors of society are required to increase brand awareness and reputation, expand international markets, and enhance the global competitiveness of agricultural products. To achieve these goals, several key steps should be taken. First, the focus should be on Miwu product innovation and quality improvement to enhance product value and competitiveness, attracting consumer attention and recognition. Second, building a strong brand image is essential, which can be achieved by creating a unique brand image through visual design and word-of-mouth communication, thereby enhancing brand reputation and recognition. Third, expanding into international markets requires international trade and cooperation to increase the global influence and popularity of agricultural products. Fourth, participating in international agricultural exhibitions and exchange activities promotes agricultural product brands and high-quality products while enhancing international communication and cooperation. Finally, improving the processing technology of products is crucial to constantly improve product quality while adding product function and value to meet the needs and competition of domestic and foreign markets.

#### 7.5.7. Improving the Business and Market System

Firstly, there should be more training for cooperatives and Miwu brokers, and business cooperation models such as “farmers + cooperatives + companies” and “farmers + brokers + markets” should be promoted. Secondly, the regulation of related products should be strengthened to strictly control the production, processing, transportation, and sales of agricultural products, ensuring their quality and safety. Thirdly, the industrialization, scale, branding, and standardization of agriculture should be promoted to upgrade the modernization of the production, processing, circulation, and sales of agricultural products, improving their added value and competitiveness. Fourthly, the sales channels of agricultural products should be expanded and diversified sales channels such as rural e-commerce, community group buying, and online sales should be developed to increase the market share and visibility of agricultural products. Fifthly, support and assistance to farmers should be strengthened to improve their production technology and management level, increase their income, and fundamentally improve the supply and quality of agricultural products in the market.

## 8. Conclusions

Miwu is a traditional medicine and food homology with a large number of resources in Sichuan, where it has been in meals for more than 800 years. Meanwhile, it has never been interrupted by the change of dynasties. Miwu is rich in carbohydrates, dietary fiber, protein, iron, zinc, nicotinic acid, and β-carotene as well as a large number of trace elements. Because of its *Daodi* characteristic, Miwu has inherent advantages in culture, religion, geographical location, infrastructure, and brand building. Increasing international influence, the continuous expansion of the domestic health market, and the good business and logistics system in the *Daodi* areas provide potential impetus for the future industrial development of Miwu. However, labor costs, government supervision, technological innovation capabilities, and vague local development schemes are becoming obstacles to the transformation and upgrading of the Miwu industry. In the future, government and social capitals should be fully attracted to carry out effective industry–university research cooperation to promote further food toxicology and hygiene research. At the same time, it is necessary to deepen the construction of food safety standardization of Miwu to make it a legal LSFRM. Additionally, promoting the value of Miwu with a wider range of development and utilization can give full play to its role in protecting food safety and nutrition.

## Figures and Tables

**Table 1:** Traditional meal usage of Miwu.

Book Name	Publication Time	Edible Part	Diet Method
*Illustration of Materia Medica*	In 1016	Leaves	Tea
*Lv Chan Yan Materia Medica*	In 1220	Leaves	Tea
*Materia Medica for Famine Relief*	In 1406	Tender leaves	Fry
*Compendium of Materia Medica*	In 1578	Leaves	Tea
*Collected Works of Materia Medica*	In 1619	Leaves	Tea
*Food materia medica*	Around 1650	Leaves, flower	Tea
*An Illustrated Book of Plants*	In 1848	Leaves	Decoction, Frying

**Table 2:** Nutritional components of Miwu.

Name	Miwu (mg/100 g)	Celery (mg/100 g)
Carbohydrate	7520	3900
Dietary fiber	5900	1400
Protein	4500	800
Fat	120	100
Na	8.27	517
P	78.9	50
Mg	31.8	10
Ca	160	152
Fe	6.26	0.8
Zn	1.35	0.46
Se	N/A	0.6 μg
Vitamin A	N/A	10 μg
Vitamin B1	N/A	0.01
Vitamin B2	0.175	0.08
Vitamin C	2.91	12
Vitamin E	0.164	2.21
Niacin	2.95	0.4
Folic acid	N/A	29
β-Carotene	1.4	0.1

The data for celery refers to China Food Composition Tables 2022 [27]. GB refers to the code name of the national standard of the determination method, relevant information can be queried at https://std.samr.gov.cn/gb/gbQuery (accessed on 7 March 2022). Carbohydrate and folic acid were detected according to GB/Z 21922-2008 (fundamental terminology and definition of the nutritional component in foods) and GB15570 (National Food Safety Standard: food additives—folic acid). The other indicators in this table were tested according to GB5009 (National Food Safety Standard). N/A means that the component cannot be detected.

**Table 3:** Chemical components of Miwu.

Code	Name	CAS	Type of Compound	Molecular Formula	Extraction Solvent	Source
1	Sabinene	3387-41-5	Terpenes and its enols	C_10_H_16_	water	[31]
2	β-Myrcene	123-35-3	Terpenes and its enols	C_10_H_16_	water	
3	α-Phellandrene	99-83-2	Terpenes and its enols	C_10_H_16_	water	
4	α-Terprinene	99-86-5	Terpenes and its enols	C_10_H_16_	water	
5	o-Cymene	524-84-4	Terpenes and its enols	C_10_H_14_	water	
6	Trans-β-Ocimenen	3779-61-1	Terpenes and its enols	C_10_H_16_	water	
7	Cis-β-Ocimenen	13877-91-3	Terpenes and its enols	C_10_H_16_	water	
8	γ-Terpinene	99-85-4	Terpenes and its enols	C_10_H_16_	water	
9	Terpinen-4-ol	562-74-3	Terpenes and its enols	C_10_H_18_O	water	
10	α-Terpineol	98-55-5	Terpenes and its enols	C_10_H_18_O	water	
11	1-Phenyl-1-pentanone	1009-14-9	Terpenes and its enols	C_11_H_14_O	water	
12	α-Copaene	3856-25-5	Terpenes and its enols	C_15_H_24_	water	
13	β-Elemene	515-13-9	Terpenes and its enols	C_15_H_24_	water	
14	Caryophyllene	13877-93-5	Terpenes and its enols	C_15_H_24_	water	
15	β-Selinene	17066-67-0	Terpenes and its enols	C_15_H_24_	water	[32]
16	E-Ligustilide	4431-01-0	Phthalide and its dimer	C_12_H_14_O_2_	water	
17	Z-3-Butylidenephthalide	551-08-6	Phthalide and its dimer	C_12_H_12_O_2_	alcohol	[33]
18	Senkyunolide-E	94530-83-3	Phthalide and its dimer	C_12_H_12_O_3_	alcohol	
19	Z-Senkyunolide-H	94596-27-7	Phthalide and its dimer	C_12_H_16_O_4_	alcohol	
20	Neoligustilide	4431-01-0	Phthalide and its dimer	C_12_H_14_O_2_	alcohol	
21	Z-Tokinolide A	112899-62-4	Phthalide and its dimer	C_24_H_28_O_4_	alcohol	
22	14,15-Dehydrocrepenynic acid monoglyceride		Phthalide and its dimer	C_21_H_34_O_4_	alcohol	[34]
23	Scopoletin	92-61-5	Phthalide and its dimer	C_10_H_8_O_4_	alcohol	[35]
24	Astragalin	480-10-4	Phthalide and its dimer	C_21_H_20_O_11_	alcohol	
25	Caffeic acid	331-39-5	Phenolic acid	C_9_H_8_O_4_	alcohol	
26	Daidzein	486-66-8	Others	C_15_H_10_O_4_	alcohol	
27	Aurantiamide acetate	56121-42-7	Others	C_27_H_28_N_2_O_4_	alcohol	
28	Ergosterol peroxide	2061-64-5	Terpenes and its enols	C_28_H_44_O_3_	alcohol	
29	Lignoceric acid	302912-17-0	Organic acids	C_24_H_48_O_2_	alcohol	
30	Cis-3-Hexen-1-ol	928-96-1	Terpenes and its enols	C_6_H_12_O	water	[36]
31	Z-Ligustilide	81944-09-4	Phthalide and its dimer	C_12_H_14_O_2_	water	
32	Trans-2-Hexenal	6728-26-3	aldehyde	C_6_H_10_O	water	[37]
33	Ligustilide	4431-01-0	Phthalide and its dimer	C_12_H_14_O_2_	water	
34	N-Hexadecanoic acid	57-10-3	Organic acids	C_16_H_32_O_2_	water	[31,38]
35	Tetradecanoic acid	544-63-8	Organic acids	C_14_H_28_O_2_	water	
36	Ferulic acid	1135-24-6	Phenolic acid	C_10_H_10_O_4_	water	[38]
37	Chlorogenic acid	327-97-9	Phenolic acid	C_16_H_18_O_9_	water	[39]
38	3,5-Dicaffeoyl quinic acid	89919-62-0	Phenolic acid	C_25_H_24_O_12_	water	
39	Sedanolide	6415-59-4	Phthalide and its dimer	C_12_H_18_O_2_	water	[40]
40	Tetramethylpyrazine	1124-11-4	alkaloid	C_8_H_12_N_2_	water	[41]
41	2-Propylpyridine	622-39-9	alkaloid	C_8_H_11_N	water	[7]
42	Neocnidilide	4567-33-3	Phthalide and its dimer	C_12_H_18_O_2_	water	[32]

## Data Availability

Data is contained within the article and Supplementary Material.

## Supplementary Materials

The following supporting information can be downloaded at: https://www.mdpi.com/article/10.3390/foods12071385/s1, Table S1: Some Chinese herbs approved for raw food materials.

## Nomenclature

CX	*Ligusticum chuanxiong* Hort.
CXR	*Chuanxiong Rhizoma*
Miwu	Tender stems and leaves of *Ligusticum chuanxiong* Hort.
Ch.P.	Chinese pharmacopoeia
LSFRM	Local special food raw material
