# Supplementary material for: An Underutilized Food “Miwu”: Diet History, Nutritional Evaluations, and Countermeasures for Industrial Development
Source: Foods. 2023 Mar 24;12(7):1385. doi: 10.3390/foods12071385 (PMC10093453; doi:10.3390/foods12071385)
Supplement: Supplementary file 1 [file foods-12-01385-s001.zip › foods-2247235-supplementary.pdf]

Supplementary Materials

Table S1. Some Chinese herbs approved for raw food materials

Botanical of plant	Medicinal part	Edible part	Description	Year of approval
Arctium lappa L.	dried ripe fruit	seeds and roots	The root is ordinary food, and the seed is the raw material of health food	2013
Forsythia suspensa (Thunb.) Vahl	dried ripe fruit	leaves	LSFRM	2017
Panax notoginseng (Burk.) F. H. Chen	dried roots and rhizomes	flowers, stems, roots	LSFRM	2017
Acanthopanax senticosus（Rupr.etMaxim.）Harms	dried roots and rhizomes	Fresh leaves	LSFRM	2019
Ophiopogon japonicus (L.f) Ker-Gawl.	dried tubers	Dried fibrous root	LSFRM	2019
Alisma orientale (Sam.)Juzep./*Alisma plantago-aquatica* Linn.	dried tubers	tubers	LSFRM	2019
Eucommia ulmoides Oliv.	dry bark	male flowers, leaves, seeds	Male flowers and seeds are new food raw materials, and leaves are medicine and food homologies	2019
Panax ginseng C. A. Mey.	dried roots and rhizomes	Roots, rhizomes and tissue cultures, leaves, fruits	Roots, rhizomes and tissue cultures are new food raw materials; leaves and fruits are health food raw materials	2002

*Data is collected on the NHC’s website http://www.nhc.gov.cn/ (accessed on 1 December 2022).
